# Exploring influencing factors on fall prevention intentions among nursing assistants: a qualitative study

**DOI:** 10.3389/fpubh.2026.1823811

**Published:** 2026-04-22

**Authors:** Yueting Huang, Weilin Zhang, Suqing Wang

**Affiliations:** 1 Department of Nursing, West China Tianfu Hospital, Sichuan University, Chengdu, Sichuan, China; 2 School of Nursing, Wuhan University, Wuhan, Hubei, China

**Keywords:** falls, nurse management, nursing assistants, patient safety, qualitative study, risk management

## Abstract

**Objective:**

Guided by the Theory of Planned Behavior (TPB), this study explored key factors influencing fall prevention intentions among nursing assistants in Chinese tertiary hospitals.

**Methods:**

A qualitative descriptive study was conducted. Twenty nursing assistants from a tertiary hospital in China were recruited via purposive sampling. Semi-structured interviews were analyzed using thematic analysis, with reporting guided by the COREQ checklist.

**Results:**

Three core themes consistent with the TPB emerged: (1) Ambivalent behavioral attitudes, characterized by a disconnect between valuing fall prevention and assuming responsibility, and conditional proactivity; (2) Conflicted subjective norms, including situational compromises among institutional, family, and peer expectations, and a punitive feedback culture; (3) Limited perceived behavioral control, challenges in complex situations and structural resource constraints (time, equipment, support).

**Conclusion:**

Nursing assistants' fall prevention intentions are shaped jointly by ambivalent attitudes, conflicting norms, and limited control. Multi-level interventions empowering nursing assistants, fostering a constructive organizational culture, and optimizing resources are needed to enhance their intentions and translate policy into consistent bedside practice.

## 1 Introduction

A fall is commonly defined as an event that results in an individual coming to rest inadvertently on the ground, floor, or other lower level (1). For older adults, falls are associated with significant risks of injury (2). Approximately 684,000 deaths each year are attributed to falls (3). In hospital settings, falls are among the most frequently reported adverse events, and the incidence of inpatient falls is approximately 3–5 per 1,000 bed-days (4). Studies have demonstrated that one-quarter to two-fifths of inpatient falls are associated with physical harm, and even lead to severe injuries including fractures and intracranial bleeding (5). In addition to physical injuries, falls may lead to profound psychological consequences such as fear of falling, anxiety, and loss of confidence. These consequences are found to impair patient recovery and quality of life (6), while financial, operational, and reputational burdens are imposed on healthcare systems (7).

Historically, fall prevention research and interventions have pre-dominantly centered on nurse-led risk assessment and patient-level factors (8). Commonly examined factors include advanced age, polypharmacy or inappropriate medication use, impaired balance, functional limitations, cognitive deficits, and a history of previous falls. However, contemporary healthcare delivery models are undergoing substantial transformation worldwide. Two converging trends are reshaping the landscape of inpatient care. First, global population aging has led to a surge in hospitalized older adults with complex care needs, including mobility impairment and high fall risk (9). Second, concurrent nursing shortages and cost-containment pressures have compelled healthcare systems to reconfigure their workforce (10, 11). In response, nursing assistants (also known as healthcare support workers or auxiliaries) are increasingly relied upon to deliver prolonged bedside care, including mobility assistance, toileting, and patient surveillance. This workforce redesign has positioned nursing assistants as critical, yet often overlooked, actors in inpatient fall prevention.

China is facing the rapid aging of its population, with the proportion of adults aged 60 and above expected to reach 28% by 2040 (12), and traditional family-led bedside care has declined due to smaller family sizes, rising female labor participation, and increased geographic mobility. In response to this care gap and the imperative to improve professional care quality, the Chinese government launched a national pilot program promoting “unaccompanied hospitalization” services in 2025 (13). This policy formally delegates continuous bedside responsibility—including fall prevention tasks—from families to formally trained nursing assistants, positioning them as the primary frontline caregivers in inpatient wards.

In the Chinese healthcare system, a clear distinction exists between the scopes of practice for nursing assistants and registered nurses. Under the authorization and supervision of registered nurses, nursing assistants primarily provide direct, non-invasive bedside care, assisting patients with activities of daily living, including feeding, bathing, toileting, dressing, and mobility. In fall prevention, nursing assistants are not authorized to conduct formal fall risk assessments, adjust care plans, or make independent clinical judgments, as these responsibilities are solely undertaken by registered nurses. Within their professional scope, nursing assistants exercise meaningful behavioral autonomy and implementation autonomy, manifested in task prioritization, interpretation of implementation methods, and patient interaction approaches. Examples include adjusting monitoring frequency, proactively providing safety reminders, determining when to report risks, and consistently executing basic safety precautions. These autonomous actions do not strictly adhere to scripts or mechanical instructions but genuinely reflect individual variations in decision-making intentions and operational autonomy among nursing staff. Emerging evidence suggests that, when appropriately trained and integrated, they can significantly contribute to structured fall-prevention education and risk identification (14, 15).

Despite their growing prominence, nursing assistants globally face multiple challenges. These include structural barriers such as an aging workforce, limited formal education (with most having only completed junior high school in China), inconsistent training quality, high turnover, and inadequate organizational support (16, 17). Critically, these tangible barriers are often compounded by intangible socio-professional challenges. Nursing assistants are frequently integrated into the professional frontline nursing team, yet their professional value is often overlooked. (18). Although previous studies have confirmed the significant value of nurse-led fall prevention, there is a lack of empirical research focusing on nursing assistants. As nursing assistants are increasingly becoming a core force in fall prevention for hospitalized patients, they are rarely included as active study subjects. Therefore, this study fills this research gap by exploring the key influencing factors of nursing assistants' behavioral intentions regarding fall prevention.

**Figure 1:**
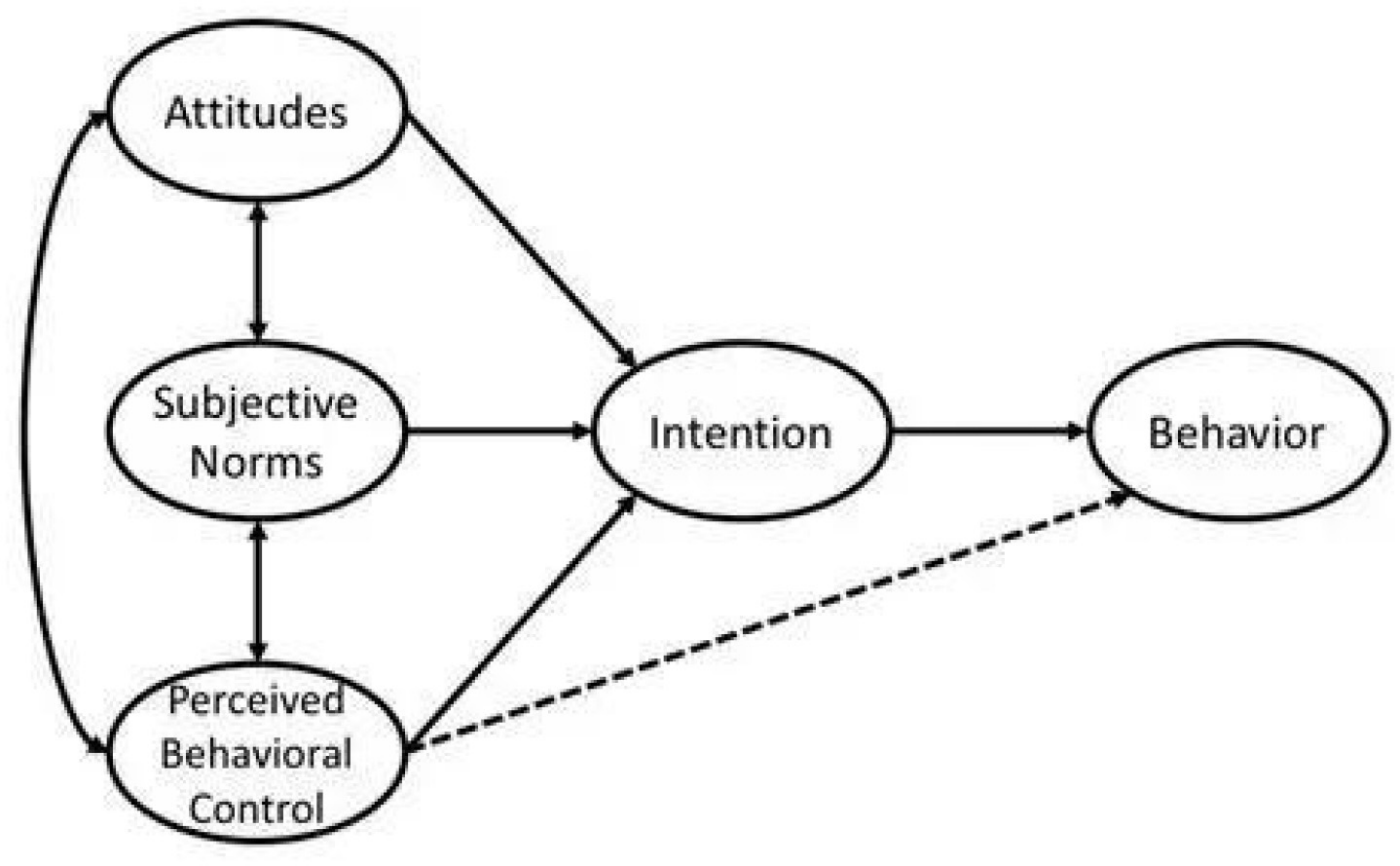
Theory of Planned Behavior model.

The Theory of Planned Behavior (TPB) was developed by Ajzen as an extension of the Theory of Reasoned Action (TRA) (19). The TRA assumed that individuals possess full volitional control over their behaviors, positing that behavior is determined solely by attitudes and subjective norms. However, Ajzen further observed that in organizational or institutional contexts, human behavior is rarely entirely voluntary; instead, it is frequently constrained by external resources, role boundaries, and managerial intervention. To address this limitation, TPB incorporated the construct of Perceived Behavioral Control, which captures an individual's subjective judgment of the ease or difficulty of performing a behavior—encompassing both internal capabilities and external constraints. This extension enables TPB to more accurately explain behavioral intention and decision-making in contexts with limited autonomy. As illustrated in Figure 1, the core constructs of TPB include: (1) Attitudes (positive or negative evaluations of the behavior); (2) Subjective Norms (perceived social pressure to perform or not perform the behavior); and (3) Perceived Behavioral Control (perceptions of the presence of resources and opportunities facilitating the behavior). These three constructs jointly shape behavioral intention, which in turn influences actual behavior. The TPB has been widely adopted in healthcare settings to explain safety-related intentions and compliance, including nurses' adherence to hospital safety protocols (20).

This study employed a qualitative descriptive design and used the TPB as a guiding framework to analyze the key factors associated with nursing assistants' fall prevention behavioral intention and to explore the specific challenges they face.

## 2 Methods

### 2.1 Aims

Guided by the TPB, this study aimed to explore the key factors associated with nursing assistants' intentions to perform fall prevention behaviors in Chinese tertiary hospital settings and the interplay between these factors.

### 2.2 Design

A qualitative descriptive approach was adopted to generate low-inference, data-driven accounts of nursing assistants' experiences and perceptions of inpatient fall prevention (21, 22). This design is well-suited to summarizing participants' perspectives in their everyday language and work context while maintaining analytical rigor. Unlike phenomenology, which focuses on the essence of lived experience, or grounded theory, which aims to develop new theoretical models, qualitative description seeks to provide a comprehensive, accessible summary of participants' perspectives. This approach aligns with the study's aim to identify practical, actionable factors influencing fall prevention intentions. The study was reported in accordance with the Consolidated Criteria for Reporting Qualitative Research (COREQ) 32-item checklist (23).

### 2.3 Participants and settings

The study was conducted in a tertiary general hospital in China. Participants were nursing assistants working in inpatient wards that required frequent bedside care, such as mobility assistance, patient transfers, and toileting support. Patient falls had been documented in these wards within the 12 months prior to data collection.

A purposive sampling strategy with maximum variation was adopted in this study to include participants with diverse characteristics, including gender, age, educational background, years of work experience, and type of ward (24). The sample size was determined by data saturation, defined as the point at which no new codes or themes emerged during iterative data collection and analysis (25, 26). Data saturation was initially achieved after 18 interviews, and two additional interviews were conducted to confirm saturation. Finally, data collection was terminated after 20 interviews, as no new themes or codes were identified.

Inclusion criteria: (1) Aged ≥18 years (According to Chinese law, individuals who have reached the age of 18 are considered adults with full legal capacity and can provide informed consent for participation in research); (2) ≥1 year of nursing assistant work experience; (3) Currently engaged in direct bedside care (mobility/transfer assistance, toileting, bathing, bedside surveillance); (4) Able to communicate clearly, willing to share work experiences, and provide written informed consent for audio-recording; (5) Hold a valid nursing assistant qualification certificate issued by a provincial health authority, indicating completion of standardized training and assessment. Exclusion criteria: (1) Obvious physical/psychological discomfort precluding interview completion; (2) Nursing assistants in managerial/administrative roles with limited routine bedside care; (3) Nursing assistants working in units with distinct workflows/fall-risk management (pediatrics, operating rooms, intensive care units, outpatient departments). All participants were anonymized and identified by numerical codes (P1–P20) to protect confidentiality.

### 2.4 Data collection

Data were collected between August and October 2025 via face-to-face semi-structured individual interviews conducted in a quiet, private room within the study hospital. Interviews were administered by two graduate students with systematic training in qualitative research methods. Neither interviewer was involved in the clinical work, supervision, or performance evaluation of the participants, which helped to minimize power differentials and encourage open communication. Interviews lasted 30–60 min, were audio-recorded with participants' written consent, and field notes were taken to capture contextual cues and non-verbal expressions. Field notes were collected immediately after each interview (within 30 min) to ensure accuracy, using a structured format including three components: (1) non-verbal behaviors of participants (e.g., facial expressions, body language, tone of voice); (2) contextual details (e.g., interview environment, participant mood during the interview); (3) researchers' preliminary reflections on key points. During data analysis, field notes were integrated with interview transcripts: non-verbal cues were used to interpret the emotional connotation of participants' verbal responses, and contextual details were used to contextualize thematic analysis, ensuring that interpretations were consistent with the participants' actual expressions and interview context.

The interview guide was developed based on the TPB framework and refined through literature review, team discussion, and two pilot interviews to optimize clarity and flow (pilot data were not included in the final analysis). The interview outline is as follows: (1) Attitude: How do you feel about preventing patient falls? What impact do you think it has on patients' health and quality of life? In your daily work, do you proactively take measures to prevent patient falls? (2) Subjective norms: What expectations do healthcare professionals, patients' relatives, and colleagues have regarding your behavior in preventing patient falls? How do you handle these expectations when they are inconsistent? How do these expectations affect your work in fall prevention? (3) Perceived behavioral control: What difficulties do you encounter in the process of preventing patient falls? Do you feel capable of competently performing various fall prevention procedures? Which external factors (such as training, equipment, or time) affect your ability to carry out fall prevention work?

Trustworthiness strategies for data collection included: (1) Brief on-site verification (interviewers summarized key points at the end of each interview and invited participants to confirm or clarify interpretations); (2) Member checking (three participants—P1, P9, and P18—reviewed preliminary thematic interpretations to confirm alignment with their experiences and intended meanings).

### 2.5 Data analysis

All audio recordings were transcribed verbatim and de-identified, with transcripts cross-checked against recordings for accuracy. Data were managed using NVivo 12 (QSR International). Thematic analysis was conducted in accordance with Braun and Clarke's six-phase approach (27): (1) Familiarization with data via repeated reading; (2) Generation of initial codes; (3) Searching for candidate themes; (4) Reviewing themes against the full dataset; (5) Defining and naming themes; (6) Producing the final report with representative participant quotations. Coding was initially conducted independently by two researchers (YH and WZ) to enhance analytical rigor. The two coders independently reviewed the transcripts, generated initial codes, and then met to compare their coding schemes. Discrepancies in coding and theme development were resolved through iterative discussion and consensus-building with the broader research team (including SW), with all decisions documented to maintain an audit trail.

### 2.6 Rigor

Multiple strategies were used to enhance the trustworthiness of the qualitative findings (28): (1) Maximum variation sampling to ensure diverse participant experiences and rich, informative data; (2) Iterative data collection and concurrent analysis to confirm data saturation; (3) Field notes to capture contextual and non-verbal cues for interpretive accuracy; (4) Regular research team discussions to minimize individual bias and reach consensus on coding/themes; (5) Member checking with three participants to validate preliminary thematic interpretations; (6) Adherence to the COREQ checklist for transparent reporting of qualitative methods and findings.

### 2.7 Ethical considerations

Ethical approval was obtained from the Ethics Committee of West China Tianfu Hospital, Sichuan University (Chengdu, Sichuan, China; Approval No.: 2025048). Written informed consent was acquired from all participants prior to data collection. Participation was voluntary, and participants were permitted to withdraw at any time without adverse consequences. All study data (transcripts, audio recordings, field notes) were anonymized and stored securely with access restricted to the research team only.

## 3 Results

A total of 20 nursing assistants were recruited, with demographic characteristics summarized in Table 1. The sample was pre-dominantly female (90.0%, *n* = 18), with a mean age of 43.05 years (SD ± 8.92), and 60.0% (*n* = 12) were aged ≤ 44 years. The minimum age of participants was 35 years, with all participants aged ≥18 years, consistent with the inclusion criterion and legal adulthood requirements in China. Most participants had a junior high school education (70.0%, *n* = 14). Professional work experience ranged from 1–9 years (mean = 4.05 years, SD ± 2.31), with 50.0% (*n* = 10) having 3–5 years of experience.

**Table 1:** Demographic and work-related characteristics of participants (*n* = 20).

Characteristics	Categories	*n*	%
Gender	Male	2	10.0
	Female	18	90.0
Age	≤ 44 years	12	60.0
	45–59 years	8	40.0
	≥60 years	0	0.0
Educational background	Primary school	1	5.0
	Junior high school	14	70.0
	Senior high school	5	25.0
Professional experience	1–2 years	5	25.0
	3–5 years	10	50.0
	6–9 years	5	25.0

**Figure 2:**
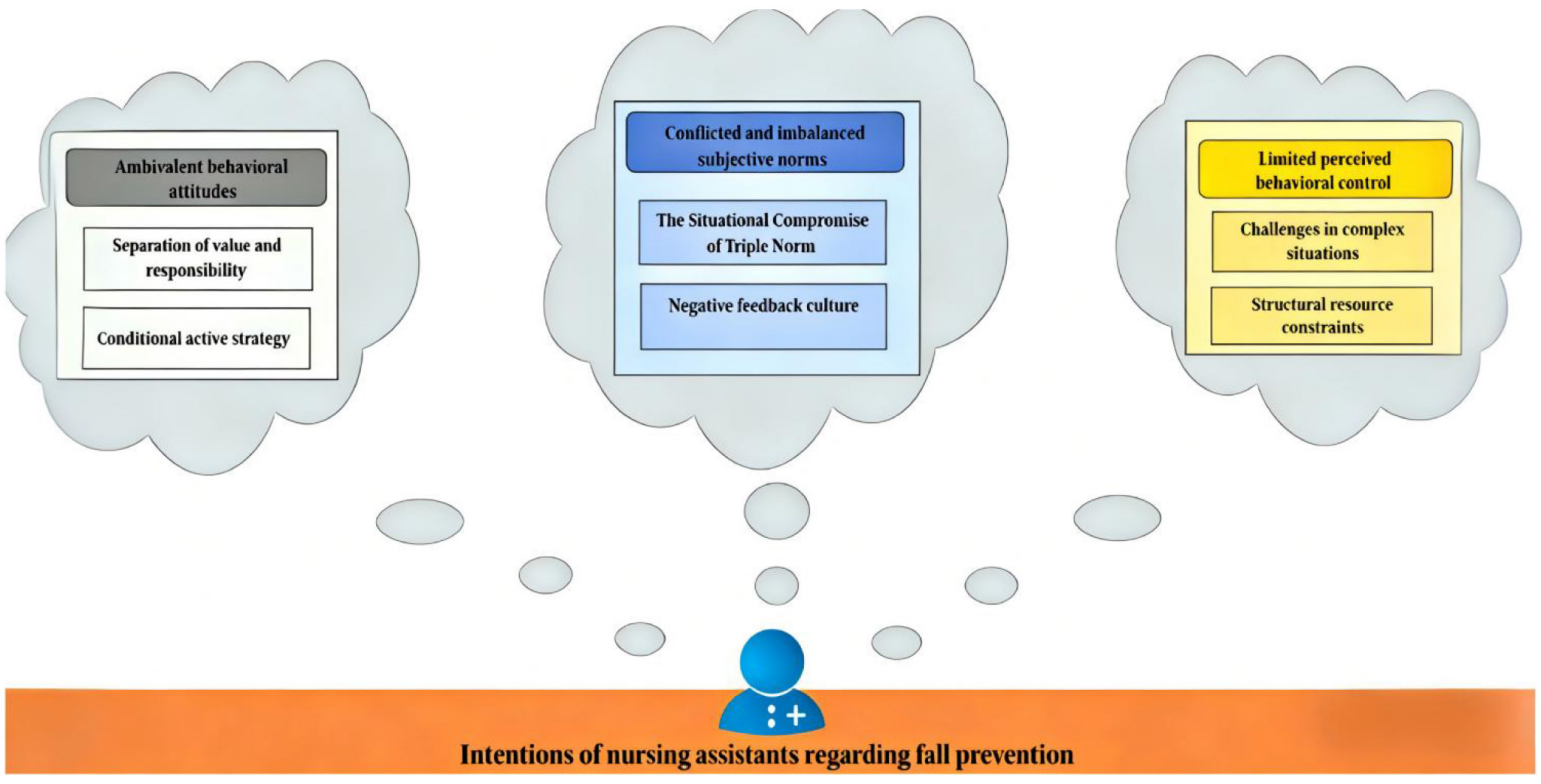
Thematic map of fall prevention intentions.

The thematic analysis identified three core themes and six sub-themes (Figure 2), all of which align with the TPB framework.

### 3.1 Ambivalent behavioral attitudes

Nursing assistants commonly described an ambivalent attitude structure toward fall prevention. While they readily endorsed the importance of fall prevention at an abstract level, this was often accompanied by a tendency to distance themselves from direct responsibility. Consequently, their motivation to engage in fall-prevention behaviors appeared externally contingent and situation-dependent.

#### 3.1.1 Separation of value and responsibility

All participants articulated a clear understanding that fall prevention is crucial for patient safety, reducing complications, and shortening hospital stays. However, when discussing responsibility attribution, many “externalized” primary responsibility to nurses, positioning themselves as passive implementers rather than accountable actors.

“*Of course, fall prevention is important—one fall could undo an older person's rehabilitation. But it's mainly nurses who assess and give instructions; we just follow their arrangements.”* (P4)

Similarly, P8 described fall prevention as compliance with institutional rules rather than an autonomous professional commitment.

“*I understand it matters for patient safety, but in my role, it feels more like following regulations than something I decide to do based on my own professional judgment.” (P8)*

This value–responsibility disconnect tended to reduce fall prevention to an externally assigned task rather than an internally endorsed core responsibility, thereby weakening intrinsic motivation. This reflects a limited sense of proactive ownership shaped by role positioning and institutional expectations.

#### 3.1.2 Conditional active strategy

Participants' proactivity was not a stable personal trait; rather, it varied with perceived situational risk and anticipated consequences. Proactivity was higher when supervision was salient (e.g., nurses were present), when operational risk was low, or when performance was likely to be recognized. In contrast, in ambiguous situations, when physical handling risk was high, or when outcomes were difficult to quantify, participants often adopted defensive passivity or risk-transfer strategies.

“*I usually refer to the nurse's assessment of the patient's risk level. If the nurse marks them as high risk, I'll be more alert and check more frequently.” (P6)*

“*When assisting patients with mobility, if the patient is very heavy or agitated, I'd rather call someone else first, because if I handle it poorly, something could go wrong.” (P11)*

### 3.2 Conflicted and imbalanced subjective norms

Nursing assistants' fall prevention behaviors were shaped by competing normative expectations, compounded by a lack of constructive feedback mechanisms. This resulted in unclear and unstable behavioral standards that shifted across situations.

#### 3.2.1 The situational compromise of triple norm

Participants consistently perceived three sources of normative pressure: institutional norms from healthcare professionals, relational norms from family members, and informal group norms from peers. These norms were implicitly prioritized depending on perceived power and immediacy. Family members' real-time requests—often as the most immediate “service recipients”—were frequently prioritized over formal ward rules, while peers' routine practices functioned as a strong informal norm.

When expectations conflicted, contextual compromise was the dominant coping strategy.

“*According to the protocol, I should go to check and remind them regularly. However, sometimes family members say: “My mother has just fallen asleep. Please do not wake her up frequently.” This situation is rather difficult, so I usually prioritize respecting the family's wishes.” (P5)*

Peer norms also exerted disciplinary influence.

“*If you are a new staff member and insist on strictly following every procedure, your colleagues may think you are rigid and not a team player.” (P10)*

Such compromises contributed to inconsistent fall-prevention practices, which fluctuated with context and who was present.

#### 3.2.2 Negative feedback culture

Organizational feedback regarding fall prevention was perceived as markedly imbalanced. Routine adherence and successful prevention were largely invisible and rarely acknowledged, creating a “feedback vacuum” in which effort was overlooked precisely because no adverse event occurred.

“*Even if I remind patients to be careful, no one encourages it. Sometimes patients even think I'm meddling.” (P3)*

In contrast, when an adverse event occurred, feedback became intense and pre-dominantly punitive, focusing on individual blame and outcomes rather than process learning or system improvement.

“*After a patient fell during nighttime toileting, I was publicly criticized for not checking enough. But no one clarified what “enough” actually means or used it as a chance to train us.” (P11)*

“*To avoid punishment, I will not actively report the problem, but instead, I will avoid blame and choose to ignore it.” (P6)*

A culture of negative feedback not only fails to improve safety, but may also encourage avoidance behaviors such as underreporting risks and minimizing exposure to avoid blame.

### 3.3 Limited perceived behavioral control

Participants generally rated their ability to carry out fall-prevention behaviors as limited. This perceived limitation stems from two aspects (1) individual-level challenges in applying knowledge to complex clinical situations, and (2) system-level resource constraints that limited their ability to act even when they possessed the necessary knowledge and skills. Together, these factors constituted major barriers to implementation.

#### 3.3.1 Challenges in complex situations

Although most participants had received basic fall-prevention training, they reported low confidence in translating static knowledge into dynamic and complex bedside contexts. Capability gaps were evident in three areas.

First, participants described limited confidence in dynamic scenarios, as training prioritized theory over simulation for complex situations.

“*Training tells us to check if the floor is wet and if bed rails are up. But in real situations, what's harder is judging the patient's condition at that moment. If they say, “I feel dizzy,” should I help them sit down immediately or is it just mild discomfort? I can't judge that well.” (P7)*

Although nursing assistants are not authorized to conduct formal clinical evaluations, they are required to report such symptoms to the supervising nurse immediately for professional assessment.

Second, participants felt difficulties in skill transition in high-demand transfer and handling scenarios, particularly with obese, agitated, or severely frail patients.

“*In theory, we're told to use transfer boards and to save effort. But when you're turning a patient who weighs over 100 kg and there aren't enough staff, the techniques don't really work—you end up using brute force. I'm very worried about injuring the patient or myself.” (P14)*

Third, participants described a lack of emergency response capacity following falls.

“*If a patient actually falls, I wouldn't dare move them because I'm afraid of causing secondary injury. But what should I observe, what should I ask, what immediate steps should I take? I don't know—I can only wait for the nurse.” (P1)*

Nursing assistants are expected to monitor, report, and await nursing support, but lack of structured training leaves uncertainty about appropriate initial responses. Several participants attributed these gaps to training formats that relied heavily on didactic teaching with limited simulation, practice, and ongoing coaching.

“*Training is usually just listening to a lecture with slides. There's no hands-on practice. When something happens, you still panic.” (P12)*

#### 3.3.2 Structural resource constraints

Even when participants expressed willingness and basic awareness, implementation was constrained by rigid external conditions.

First, time scarcity was repeatedly emphasized.

“*From morning to night, it feels like a battle. Basic care tasks are non-negotiable. Checking each bed space for hazards—if there's time, you do it; if not, you prioritize what seems most urgent.” (P9)*

Second, participants described limited access to appropriate equipment.

“*Our ward only has two transfer boards, and they're often borrowed by other wards. When there isn't one, we have to lift manually. It's unsafe and families may question what we're doing.” (P16)*

Third, participants highlighted a lack of timely professional support when uncertainty arose.

“*When I'm not sure, I want to ask the nurse, but sometimes they're busy with dressing changes or infusions and can't take time to explain.” (P13)*

## 4 Discussion

This qualitative study, grounded in the TPB framework, identifies three interrelated core themes influencing the fall-prevention intention of nursing assistants in tertiary hospitals in China: ambivalent attitudes, conflicting subjective norms, and limited perceived behavioral control. Collectively, these themes confirm the implementation gap between fall-prevention policies and actual bedside care practices (29). This study makes three fold innovative and unique contributions. First, to our knowledge, it is the first qualitative study specifically exploring the fall-prevention intention of nursing assistants, filling a gap in existing literature that primarily focuses on nurse-led fall-prevention models. Second, conducted under the context of the “unaccompanied care” policy—a unique healthcare reform initiative that formally transfers bedside fall-prevention responsibilities from family members to nursing assistants—it provides a perspective missing in international research. Third, by extending the application scope of TPB to the understudied frontline nursing group (nursing assistants), it deepens theoretical understanding of the safety behaviors of subordinates in healthcare. These findings respond to the global call to integrate nursing assistants into multidisciplinary fall-prevention systems (15, 30).

A key finding of this study was that nursing assistants exhibited ambivalence toward fall prevention. On the one hand, they generally recognized the significance of fall prevention for patient safety; on the other hand, they consistently attributed the primary responsibility for fall prevention to registered nurses. From the perspective of the TPB, this disjunction weakens behavioral intention, as it prevents nursing assistants from internalizing fall prevention as a core professional responsibility (19). China's healthcare system is characterized by clear professional hierarchies and welldefined power boundaries (31), nursing assistants are institutionally positioned as frontline care staff. This structural positioning hinders their internalization of a professional identity, as reflected in the mindset that “they (nurses) think, we execute” (18). The present findings align with van Wieringen's description of a perceived hierarchical distance between professional roles (32). Such a mentality undermines professional self-efficacy and the psychological sense of ownership over care outcomes, both of which are essential drivers of proactive safety practices.

Likewise, the conditional proactivity demonstrated by nursing assistants represents a direct behavioral manifestation of such attitudinal ambivalence. When intrinsic motivation is diminished, behavioral patterns are pre-dominantly guided by external situational cues rather than internal professional values. This finding is consistent with prior research on nurses' safety behaviors (20). China's “unaccompanied care” model (13) has further exacerbated this issue. This model has expanded the clinical responsibilities of nursing assistants without a corresponding enhancement in their professional identity or decision-making authority. As noted by Jia et al. (30), such an imbalance between responsibility and autonomy poses a significant threat to patient safety. This imbalance underscores the need to deliberately empower nursing assistants and reposition them as proactive safety partners, rather than passive task performers.

The behavioral patterns of nursing assistants in response to multiple normative pressures validate the Focus Theory of Normative Conduct proposed by Cialdini et al. (33). This theory posits that individuals will act in accordance with the most salient normative cues in a specific situation. The present study found that participants generally perceived three sources of normative pressure, specifically including institutional norms formulated by medical professionals, relational norms established by family members, and informal group norms formed by colleagues. Notably, the immediate demands of family members and informal peer norms were usually more influential than formal institutional fall prevention rules. This finding is consistent with the results of Li et al.'s (34) research on the vigilance of family caregivers in pediatric nursing, and it also confirms the role conflict encountered by nursing assistants as frontline coordinators when responding to multiple normative demands.

More concerning is the negative organizational feedback culture, which is characterized by punitive responses to errors while remaining silent on successful prevention measures. This imbalance has created a distorted incentive mechanism that not only inhibits proactive risk mitigation behaviors but also fosters defensive practices, including risk concealment (35). This pattern runs counter to global patient safety initiatives advocating a just culture, which emphasizes learning from errors rather than blaming individuals (35). Under the unaccompanied care model, the absence of family caregivers may reduce certain normative conflicts but exacerbate tensions between institutional protocols and individual practices. This dynamic makes a constructive, learning-oriented feedback culture particularly crucial.

Perceived behavioral control is a strong predictor of behavioral intention (19). In this study, perceived behavioral control faced severe limitations due to dual challenges, including deficiencies in both capabilities and resources. Although most participants had received basic fall prevention training, they encountered significant difficulties in translating static theoretical knowledge into dynamic, complex bedside scenarios encountered in daily practice. However, these challenges should not be attributed to individual shortcomings but rather recognized as consequences of inadequate systematic training and support systems. As Bandura's social cognitive theory suggests, self-efficacy is established through experiential mastery, vicarious learning, and guided feedback—elements that are systematically absent from current training frameworks for nursing assistants(36). Current training programs for nursing assistants rely on passive, lecture-based knowledge transmission rather than competency-driven skill development approaches.(17, 37). Meanwhile, structural resource constraints further undermined perceived control. Nursing assistants repeatedly cited issues such as high time pressure, insufficient equipment, and limited professional support. These findings align with those of Najafpour et al., (5) who identified resource constraints as a major barrier to safe inpatient care. For nursing assistants, who are already in a disadvantaged position in healthcare resource allocation (16), resource scarcity constitutes a typical predicament that directly inhibits their intention to prevent falls.

### 4.1 Strengths and limitations

This study has several strengths. First, it centers the understudied perspective of nursing assistants in fall prevention research. Second, it uses a rigorous qualitative descriptive design guided by the TPB and aligned with the COREQ checklist. Third, it identifies interconnected, multi-level barriers to fall prevention intention that inform actionable interventions.

The study has several limitations. First, its single-center design (only one tertiary hospital) excludes secondary hospitals, community health centers, and nursing homes in different regions of China. The degree of behavioral discretion among nursing assistants may also vary by institutional management, staffing models, nurse-assistant collaboration, and ward practices, which limiting result transferability. Second, self-report bias may exist, as data rely on nursing assistants' self-reported perceptions; despite confidentiality measures, social desirability bias may have influenced responses. Third, the cross-sectional design identifies factors affecting fall prevention intentions but does not test the causal relationship between TPB constructs and actual fall prevention behaviors.

Future research priorities include: (1) Using mixed methods to quantitatively examine the association between the TPB-based fall prevention intention model and objective behaviors in large-scale, multi-center samples; (2) Designing and evaluating multi-level interventions to verify the effectiveness and sustainability of empowerment, organizational alignment, and system resource strategies identified in this study; (3) Conducting cross-cultural comparative studies to explore how healthcare systems and cultural factors influence nursing assistants' safety behaviors, to generate more generalizable theoretical and practical guidance.

### 4.2 Policy and practice implications

The study's integrative findings indicate that isolated, single-component interventions are unlikely to improve nursing assistants' fall prevention intention or practice. Training alone, for example, would be insufficient. Instead, multi-level, synergistic strategies are required to break the cycle of vulnerability and translate fall prevention policy into consistent bedside practice. These strategies must address the interconnected TPB constructs of attitude, norms, and perceived control. They align with global patient safety and implementation science principles (37) and are tailored to the unique context of Chinese hospital care.

First, individual and team empowerment should address ambivalent attitudes and the knowing-doing gap. Fall prevention training should be reformed to shift from knowledge transmission to competency-based development. This development should center on simulation, hands-on practice, and ongoing bedside coaching. The focus should include dynamic risk assessment, high-risk patient handling, and post-fall response. Critically, training must be paired with efforts to intentionally reframe nursing assistants' professional identity. This identity should shift from “frontline care staff” to “safety partners.” The reframing involves clearly articulating their formal role, ownership, and authority in fall prevention. It also requires establishing peer recognition mechanisms, such as “Safety Champion” awards, to celebrate proactive safety practice.

Second, organizational culture and management recalibration should address conflicting norms and the negative feedback culture. Healthcare institutions should proactively align normative expectations for fall prevention across all stakeholders. This alignment requires structured communication, including daily team huddles that include nursing assistants and family education sessions upon admission. Peer support groups should be established, and “fall prevention champions” should be designated among nursing assistants to cultivate positive informal norms. Fundamentally, the organizational feedback culture must be reformed to establish a just, learning-oriented system. Such a system makes proactive fall prevention behaviors visible and recognized. When adverse events occur, the focus should be on system improvement rather than individual blame.

Third, systemic resourcing of frontline practice should address structural resource deficiencies. Workflow redesign should be implemented to allocate dedicated time for nursing assistants to perform key fall prevention actions. These actions include environmental checks and patient education. The goal is to create “protected time” for safety without compromising basic care tasks. Consistent access to well-maintained assistive equipment must be ensured. Finally, rapid, non-judgmental clinical consultation channels should be established. These channels could include a dedicated nurse mentor or a “safety hotline” for nursing assistants to address uncertainty in real time. Such resources fundamentally enhance the feasibility of fall prevention practice and strengthen nursing assistants' perceived behavioral control.

## 5 Conclusion

Nursing assistants play an increasingly critical role in inpatient fall prevention amid China's “unaccompanied hospitalization” policy reform, yet their fall prevention intentions are constrained by ambivalent attitudes, conflicting subjective norms, and limited perceived behavioral control. Addressing these factors requires multi-level synergistic interventions that empower nursing assistants, reshape organizational culture, and optimize systemic resources. By doing so, healthcare institutions can enhance nursing assistants' engagement in fall prevention practices, translating policy goals into consistent, high-quality bedside care. This study not only advances our understanding of frontline fall prevention workforce but also provides a foundation for developing targeted interventions to improve patient safety in Chinese hospitals and beyond.

## Data Availability

The raw data supporting the conclusions of this article will be made available by the authors, without undue reservation.
